# Catcher Domains as Multifunctional Fusion Modules for Soluble Expression, Covalent Coupling, and Spatial Organization of Recombinant Proteins

**DOI:** 10.1002/smsc.70328

**Published:** 2026-06-24

**Authors:** Ruxia Fan, Xing Wan, Safoorah Khanum, Juhani P. Jokio, Nea B. Möttönen, Miia R. Mäkelä, A. Sesilja Aranko

**Affiliations:** ^1^ Department of Bioproducts and Biosystems School of Chemical Engineering Aalto University Espoo Finland

**Keywords:** Catcher/Tag, enzyme immobilization, liquid–liquid phase separation, protein conjugation

## Abstract

Fusion tags are essential tools for recombinant protein production, but their functions are often limited to improving solubility or facilitating purification. Catcher domains are split‐protein fragments derived from bacterial adhesion proteins that spontaneously form irreversible isopeptide bonds with their complementary Tag. Here, we describe Catcher domains as multifunctional fusion partners that not only enhance soluble protein expression but also enable modular and covalent protein assembly. Fusion of Catcher domains to diverse recombinant proteins, including both structural proteins and enzymes, remarkably enhanced their soluble expression in *Escherichia coli*. The covalent reaction of Catcher‐Tag pairs further provided a versatile platform for downstream modular protein engineering, allowing selective and oriented enzyme immobilization for purification and improved stability, covalent protein polymerization, and selective recruitment into compartmentalized environments. These proof‐of‐concept studies establish Catcher domains as multifunctional fusion tags that bridge soluble protein expression with modular covalent assembly and immobilization, offering a versatile strategy for the engineering of functional protein systems for applications in biotechnology.

## Graphical abstract

Catcher domains function as highly versatile fusion tags that simultaneously enhance soluble protein expression and enable modular protein assembly. Spontaneous, irreversible covalent coupling by Catcher‐Tag pairs provides a platform for downstream modular engineering, enabling oriented enzyme immobilization, protein polymerization, and selective compartmentalization. This versatile platform enables bridging upstream production with downstream protein assembly, advancing the development of advanced protein‐based systems.

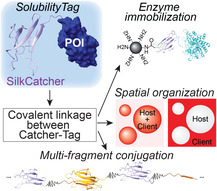

## 1 Introduction

Proteins have been widely used as building blocks for engineering functional biomaterials. Their programmable and diverse properties make them an ideal platform for the development of bio‐based materials, with applications ranging from enzymatic catalysis and biosensing to the construction of smart materials [1, 2]. However, the efficient production of soluble and functional recombinant proteins can still be challenging in many cases. Aggregation and misfolding, that lead to reduced yield and loss of activity, are key challenges in the heterologous production of recombinant proteins [3, 4, 5]. In addition, the subsequent processes that are required for the biotechnological applications of proteins, such as purification, immobilization, and assembly, depend on additional chemical modifications or multistep procedures, which may complicate the overall workflow.

A variety of fusion tags have been developed to enhance heterologous protein expression and facilitate downstream processing. Solubility‐enhancing protein domains, such as maltose‐binding protein [6, 7, 8], glutathione‐S‐transferase (GST) [9, 10, 11], thioredoxin (Trx) [7, 12, 13], and small ubiquitin‐related modifier (SUMO) [8, 14, 15], can improve the solubility and stability of aggregation‐prone proteins and facilitate purification in some cases. While affinity or detection tags like His‐tag [16, 17, 18, 19, 20], FLAG‐tag [19, 20, 21], Avi‐tag [22], and Halotag [23, 24] are mainly used for purification or labeling purposes, they have minimal impact on solubility enhancement. None of the conventional fusion tags contributes to the assembly of partner proteins. Although in certain instances the tags can be maintained and integrated with the fusion partner to preserve solubility and functionality during downstream characterization, the removal of solubility tags typically requires additional cleavage steps [25]. Expanding the toolkit of fusion partners by developing tags that can simultaneously enhance soluble expression and serve as modules for downstream assembly or functionalization would facilitate straightforward protein production and processing for biotechnological applications.

Recent advances in biomolecular click‐reaction systems have introduced powerful tools for the conjugation of genetically encoded protein [26, 27, 28, 29]. Biomolecular click reactions are based on Catcher‐Tag pairs that can form spontaneous and specific isopeptide bonds under mild conditions without external catalysts or reagents [26, 27, 28]. The irreversible linkage between Catcher‐Tag pairs has been adopted for multienzyme complexes [30, 31], modular vaccines [32], and protein‐based materials [33]. Several orthogonal crosslinking Catcher‐Tag pairs, for instance, SpyCatcher/SpyTag and SilkCatcher/SilkTag, have been developed for multimeric protein conjugation [26, 34, 35]. Moreover, genetically engineered SpyCatcher variants, which do not catalyze isopeptide bond formation, SpySwitch [36] and SpyDock [37], have been developed for affinity purification. However, the potential of Catcher domains to act as solubilizing partners and multifunctional assembly modules has been underexplored.

**FIGURE 1:**
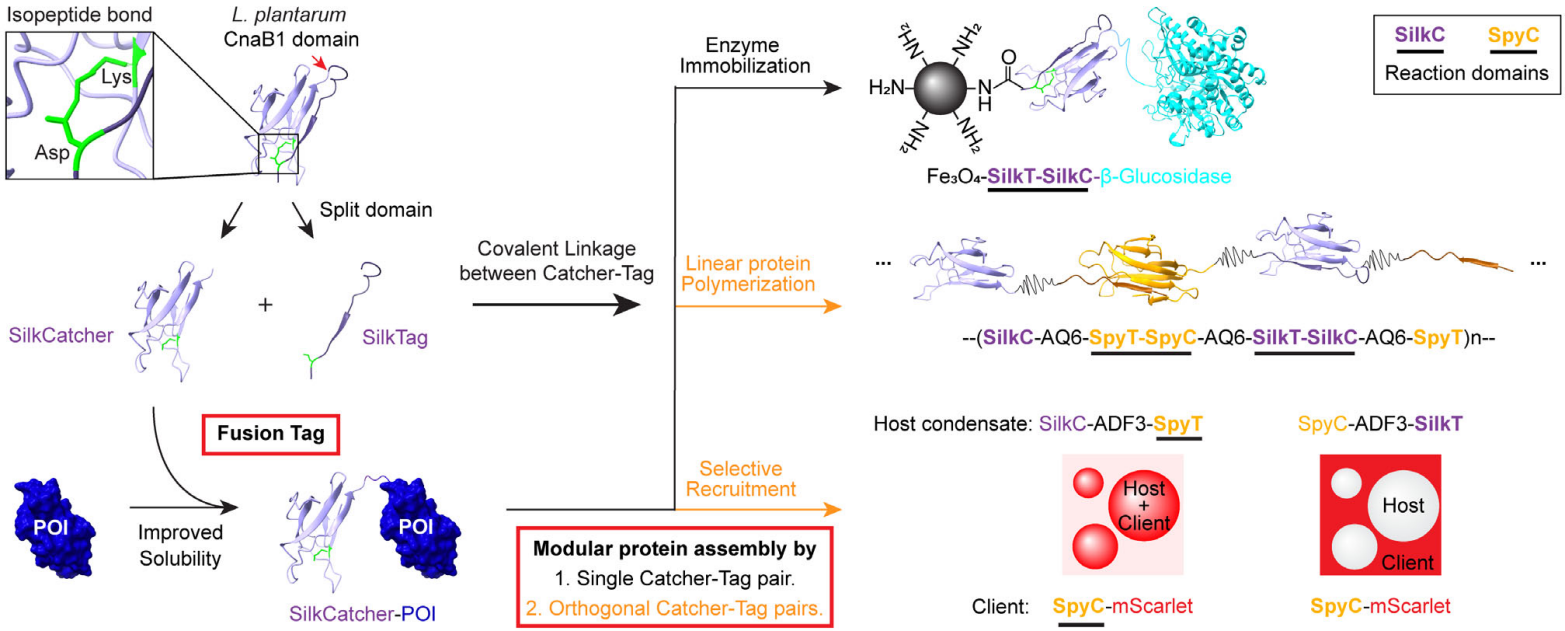
Schematic presentation of the multifunctional Catcher domain facilitates soluble heterologous protein expression and modular protein assembly, including enzyme immobilization, linear protein polymerization, and selective recruitment into compartmentalized environments. Cartoon presentation of *L. plantarum* CnaB1 domain is a homology model [34]. SilkCatcher (purple) and SilkTag (dark purple) regions and isopeptide bond (green) are shown. POI stands for protein‐of‐interest. ADF3 represents a wild type spidroin repetitive sequence from *Araneus diadematus* Fibroin‐3, AQ6 is a truncated variant of ADF3, SpyC and SpyT stand for SpyCatcher002 and SpyTag, respectively.

Here, we report that Catcher domains can serve as multifunctional fusion partners that integrate soluble protein expression enhancement with downstream covalent assembly. Fusion of Catcher domains significantly improved the soluble expression of recombinant proteins, including disordered proteins; a spider‐silk like protein and bovine caseins, as well as enzymes; β‐glucosidase and laccase. Furthermore, we demonstrate the applications of subsequent covalent crosslinking in three distinct cases. First, we show that a covalent conjugation reaction mediated by the Catcher‐Tag enables efficient immobilization of β‐glucosidase. Second, the orthogonal crosslinking reaction among different Catcher‐Tag pairs is demonstrated to allow the polymerization of spider‐silk like proteins. Finally, we show that the orthogonality of different Catcher‐Tag pairs enables the selective recruitment of target proteins into condensates (Figure 1). These findings demonstrate the potential of Catcher domains as versatile building blocks for constructing functional and programmable protein assemblies and as tools in enzyme technology and cell biology.

## 2 Results and Discussion

### 2.1 Catcher Fusion Partner Enhances Soluble Expression of Recombinant Proteins

We previously identified a SilkCatcher/SilkTag pair derived from a CnaB1 domain of a cell surface protein from *Lactobacillus plantarum* [34] (Figure 1), which showed potential as a fusion tag for enhancing protein solubility and thermal stability. To further investigate the solubility‐enhancing potential of the Catcher domain, we selected several aggregation‐prone proteins that are of biotechnological interest to produce as fusion proteins with different solubility tags.

**FIGURE 2:**
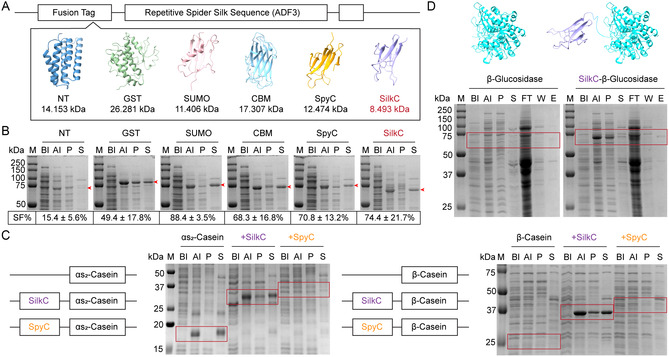
Fusion tags facilitate soluble expression of recombinant proteins. (A) Schematic presentation of the designed spider‐silk fusion proteins and cartoon structure model of different fusion tags. NT (PDB ID: 3LR2) [45], GST (PDB ID: 1B8X) [41], SUMO (PDB ID: 1L2N) [42], CBM (PDB ID: 1NBC) [43], SpyC (PDB ID: 4MLI) [46]. (B) Expression test and soluble fraction analysis of recombinant spider‐silk like proteins fused with different fusion tags. ADF3 represents a wild type spidroin repetitive sequence from *Araneus diadematus* Fibroin‐3, NT represents for N‐terminal domain from *Euprosthenops australis* MaSp1, GST represents for Glutathione S‐transferase, SUMO represents for small ubiquitin‐related modifier, CBM represents for cellulose binding domain, SpyC represents for SpyCatcher002, and SilkC represents for SilkCatcher. The red arrows indicate the position of the target protein band. SF% stands for the soluble target protein fraction of the whole target protein, data show the mean ± SD from triplicate experiments. (C) Expression test of bovine α_s2_‐casein and β‐casein fused with SilkC and SpyC. The red box stands for the region of the target protein band. (D) Expression test of β‐glucosidase fused with SilkC. β‐glucosidase (PDB ID: 3AIU) [47]. The red box indicates the (expected) region of the target protein band. M stands for marker, BI stands for whole cell before induction, AI stands for whole cell sample after induction, P stands for pellet after cell lysis, and S stands for supernatant after cell lysis. FT stands for flow‐through sample, W stands for wash, and E stands for elution. Theoretical molecular weights of six recombinant spider‐silk like proteins: NT‐ADF3‐SpyT is 62.9 kDa, GST‐ADF3‐SpyT is 75.1 kDa, SUMO‐ADF3‐SpyT is 60.1 kDa, CBM‐ADF3‐SpyT is 66.2 kDa, SpyC‐ADF3‐SilkT is 62.3 kDa, and SilkC‐ADF3‐SpyT is 57.4 kDa. Theoretical molecular weights of six casein proteins: α_s2_‐casein is 18.9 kDa, SpyC‐α_s2_‐casein is 31.3 kDa, SilkC‐α_s2_‐casein is 27.5 kDa, β‐casein is 25.3 kDa, SpyC‐β‐casein is 37.7 kDa, and SilkC‐β‐casein is 33.9 kDa. Theoretical molecular weight of β‐glucosidase is 61.4 kDa and SilkC‐β‐glucosidase is 68.8 kDa.

A 43 kDa fragment of a wild type spidroin repetitive sequence from *Araneus diadematus*, ADF3 [38], was the first model protein tested in this work. The recombinant production of spider‐silk like proteins is of interest for applications in biomaterials due to the exceptional mechanical properties of spider silk. However, silk proteins are long, repetitive, and disordered in solutions, which makes them challenging to produce in high yields and soluble form [39]. In order to compare different fusion tags, we fused ADF3 with the N‐terminal domain (NT) from *Euprosthenops australis* MaSp1 [40] to construct NT‐ADF3‐SpyT. Several commonly used fusion tags, Glutathione S‐transferase (GST) [41], small ubiquitin‐related modifier (SUMO) [42], cellulose binding domain (CBM) [43], and the widely used Catcher domain, SpyCatcher002 (SpyC) [44], as well as SilkCatcher (SilkC) [34] were also fused with ADF3 to construct GST‐ADF3‐SpyT, SUMO‐ADF3‐SpyT, CBM‐ADF3‐SpyT, SpyC‐ADF3‐SilkT, SilkC‐ADF3‐SpyT (Figure 2A, Table S1).

We produced all fusion proteins in the same conditions and compared the expression levels and solubilities. To evaluate the solubility enhancement ability of different fusion tags, the relative percentages of the soluble fractions of target proteins were calculated based on the band intensity of SDS‐PAGE gel. (Figure 2B). Although the native NT has been reported to maintain the solubility of spider silk proteins in vivo within the spider gland [48, 49], in the recombinant construct NT‐ADF3‐SpyT, the NT domain failed to improve the soluble expression of ADF3 in *E. coli*, with only ∼15.4% soluble fraction detected. (Figure 2B). Fusion with GST domain resulted in a noticeable increase in the soluble fraction of ADF3, though approximately half of the expressed protein still presented as inclusion bodies. In contrast, fusion with SUMO, CBM, SpyC, and SilkC domains effectively enhanced the soluble expression of ADF3, yielding over ∼70% proteins in soluble form. It is worth noting that the molecular weight of SilkC is only 8.5 kDa, making it smaller than other fusion tags tested here.

In addition, the effect of different fusion tags on the thermal stability of ADF3 was further explored, except for the NT‐ADF3‐SpyT that could not be analyzed due to its poor soluble expression level. The soluble fractions of the ADF3 fusion proteins were incubated at 70°C, 80°C, and 90°C for 10 min, followed by separation of soluble and insoluble fractions. We found that ADF3 fused with GST or CBM tended to aggregate upon heating, resulting in a substantial loss of soluble protein at elevated temperatures. In contrast, SUMO‐ and Catcher‐fused ADF3 remained largely soluble even after incubation at 90°C (Figure S1). These results not only demonstrate the stabilizing effect of the SUMO and Catcher domains on fusion proteins but also suggest a potential heat‐based protein purification strategy for thermally stable proteins, in which the contaminants can be selectively removed while the target protein remains soluble under high temperature.

To further evaluate the generality of the two Catcher domains as solubility‐enhancing tags, we fused SpyC and SilkC with bovine α_s2_‐casein and β‐casein [50], respectively, and analyzed the soluble expression of casein proteins with and without Catcher fusion tags in *E. coli*. Compared with SpyC, fusion with SilkC resulted in markedly higher levels of soluble expression for both α_s2_‐casein and β‐casein (Figure 2C). We further fused the SilkC domain with two enzymes, β‐glucosidase isolated from rye (*Secale cereale*, cv. Motto) [51] and laccase from the white‐rot fungus *Panus rudis* (protein ID: 1 594 824 in JGI MycoCosm, https://mycocosm.jgi.doe.gov/Panru1/Panru1.home.html [52], to evaluate the potential of SilkC to improve the recombinant expression of enzymes that play critical roles in biomass degradation and several biotechnological applications including the production of biomaterials and biochemicals [53]. To demonstrate that the SilkC fusion strategy is applicable beyond *E. coli* and intracellular expression systems, the β‐glucosidase and SilkC‐β‐glucosidase were expressed in *E. coli*, while the laccase constructs were expressed as a secreted form in *Pichia pastoris*, which preserves the posttranslational modifications and copper incorporation required for laccase activity [54]. Compared to expression without a solubility tag, fusion with SilkC exhibited a higher overall expression level of β‐glucosidase, although approximately 83% of the protein remained in the insoluble fraction (Figure 2D). The SilkC‐laccase fusion protein was successfully expressed and secreted in *P. pastoris*, but its relative catalytic activity was reduced to ∼12% of the wild‐type laccase, likely due to the N‐terminally fused SilkC affecting the yield of the secreted laccase (Figure S2A,B). The conjugation reaction between SilkC‐laccase and SilkT‐ADF3‐SpyC was confirmed by SDS‐PAGE and Western blot, demonstrating that the SilkC domain remains its conjugation functionality in the context of enzyme fusion proteins secreted from *P. pastoris* cells (Figure S2C). Overall, the SilkC fusion strategy is compatible with both intracelular expression in *E. coli* and secretory expression in *P. pastoris*; however, the fusion may affect the activity of individual enzymes, as observed for SilkC‐laccase, highlighting that optimization of the fusion construct may be required for each target protein and expression system.

We also conducted a preliminary test of SilkC as a potential purification tag. An inactive SilkT variant, lacking isopeptide bond formation activity, was immobilized on a Ni‐NTA column via a His‐tag, and SilkC‐fused proteins were captured through the specific structural affinity between SilkC and SilkT. This approach yielded the target protein with a purity exceeding 95%, although partial loss of the product was observed (Figure S3). Previously developed SpyDock [37] and SpySwitch [36], variants from SpyCatcher, have been successfully used for affinity purification. Although we did not further optimize SilkC for improving purification efficiency, these results suggest that optimization of SilkC for applications as a purification tag should be feasible.

Overall, these findings showed the potential of the Catcher domain, especially SilkC, to act as a solubility‐enhancing tag for the heterologous expression of recombinant proteins and a promising candidate for a purification tag. The retained biochemical activity of Catcher domains after fusion further expands their potential applications in covalent coupling, such as enzyme immobilization, protein polymerization, and spatial organization of proteins.

### 2.2 Catcher‐Tag Covalent Coupling Enables Enzyme Immobilizations

**FIGURE 3:**
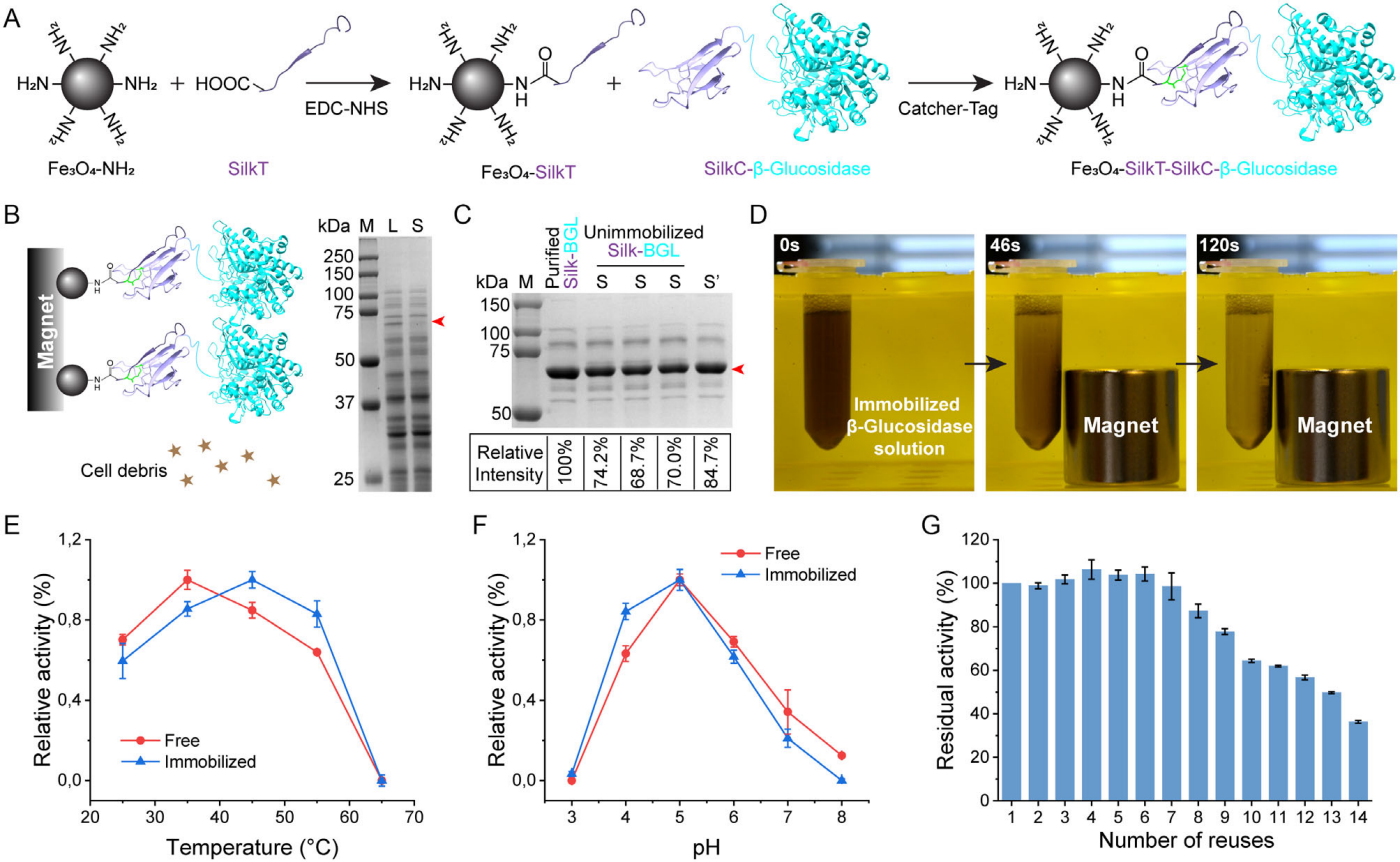
β‐glucosidase immobilization through covalent coupling via Catcher‐Tag. (A) Schematic presentation of the immobilization of β‐glucosidase on magnetic nanoparticles. (B) Catcher‐Tag‐mediated affinity purification and immobilization of β‐glucosidase. M stands for marker, L stands for cell lysate, and S stands for cell lysate after immobilization. (C) SDS‐PAGE analysis of immobilization of purified SilkC‐β‐glucosidase. M stands for marker, SilkC‐BGL stands for SilkC‐β‐glucosidase, S stands for leftover SilkC‐β‐glucosidase after immobilization with SilkT‐modified Fe_3_O_4_ nanoparticles, and S’ stands for leftover SilkC‐β‐glucosidase after immobilization with unmodified Fe_3_O_4_ nanoparticles. Relative band intensity was analyzed by ImageLab software (Bio‐Rad). (D) Photograph of the separation of immobilized β‐glucosidase via magnet. (E) Temperature dependent activity of free and immobilized β‐glucosidase. (F) pH dependent activity of free and immobilized β‐glucosidase. (G) Recyclization of immobilized β‐glucosidase. Error bars represent standard deviations of replicate experiments, (*n* = 3).

To expand the application range of the Catcher fusion tag, we took advantage of the highly specific, spontaneous, and stable covalent isopeptide bond formation between the Catcher domain and Tag peptide for enzyme immobilization. Amine‐functionalized Fe_3_O_4_ nanoparticles were first decorated by SilkT peptides using EDC‐NHS reaction, followed by reaction with SilkC‐β‐glucosidase, resulting in covalent immobilization of the β‐glucosidase (Figure 3A).

Both unpurified and purified SilkC‐β‐glucosidase could be immobilized by simply mixing with SilkT peptide decorated Fe_3_O_4_ nanoparticles (Figure 3). When SilkT‐modified nanoparticles were incubated with *E. coli* lysates containing SilkC‐β‐glucosidase, the SilkC‐β‐glucosidase was specifically immobilized and could be purified by magnetic separation, while other proteins remained in the solution (Figure 3B,D). SDS‐PAGE analysis indicated that the purified SilkC‐β‐glucosidase was successfully immobilized, with covalent attachment mediated by the Catcher‐Tag reaction, despite the presence of some nonspecific adsorption (Figure 3C). The activity of free and immobilized enzymes was tested under different temperatures and pH. The free β‐glucosidase showed maximum activity at 35°C, while the immobilized enzyme reached its peak at 45°C. Across different pH values, the relative activity of immobilized β‐glucosidase was comparable to that of the free enzyme, with slightly higher activity at pH 4.0 (Figure 3E,F). Moreover, the immobilized β‐glucosidase retained 49.7 ± 0.4% of its initial activity after 12 reuse cycles, demonstrating excellent reusability (Figure 3G).

Compared with conventional chemical immobilization methods, such as glutaraldehyde crosslinking or EDC‐NHS coupling [55, 56, 57], the Catcher‐Tag‐based immobilization provides selectivity and site‐specificity, enabling the targeted immobilization of SilkC‐fused proteins under mild conditions even in the presence of crude lysates. These results highlight the potential of the Catcher domain as a multifunctional fusion tag for selective immobilization.

### 2.3 Polymerization of Spider‐Silk Like Protein Through Orthogonal Catcher‐Tag Pairs

The selectivity of the Catcher domain is not limited to discriminating between proteins that lack the corresponding domain. Even structurally similar Catcher‐Tag pairs, such as SpyC/SpyT and SilkC/SilkT, exhibit no cross‐reactivity [34]. The orthogonal reactivity between SpyC/SpyT and SilkC/SilkT allows multiple Catcher‐Tag systems to perform modular protein assembly within the same reaction system. For instance, we previously successfully linked recombinant spider‐silk like protein monomers of around 65 kDa into multimeric, native‐sized recombinant spider‐ silk like proteins of around 320 kDa, by utilizing these two orthogonal Catcher‐Tag pairs [34]. High‐molecular‐weight spider‐silk like proteins are reported to be essential for producing superior recombinant spider silk fibers in some cases [58, 59]. To this end, we employed the orthogonal Catcher‐Tag pairs here to produce ultrahigh‐molecular‐weight silk proteins, which were subsequently processed into recombinant spider silk fibers through wet spinning methods we developed before [60].

**FIGURE 4:**
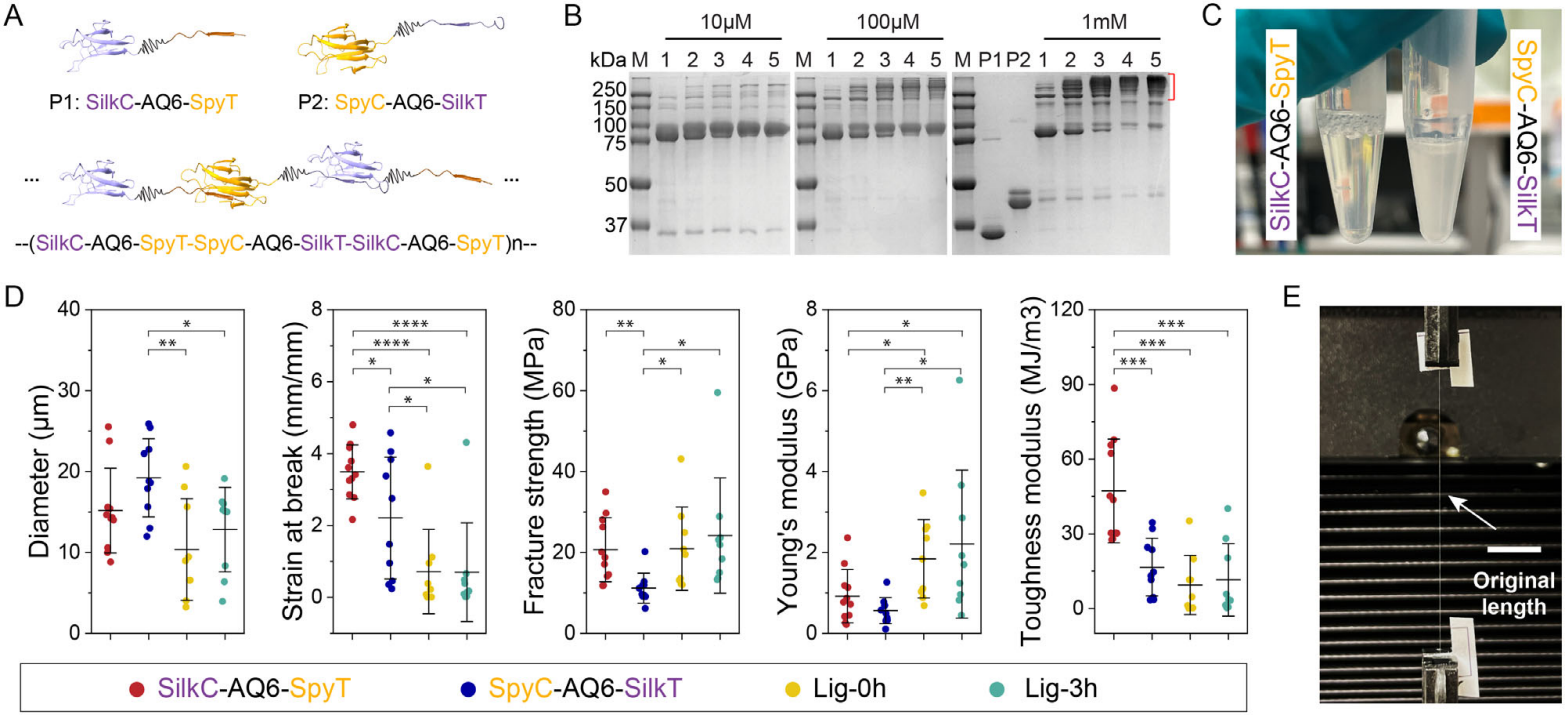
Polymerization of recombinant spider‐silk like proteins mediated by orthogonal Catcher‐Tag pairs. (A) Schematic presentation of polymerization. (B) SDS‐PAGE images of polymerization under different precursor concentrations. M stands for marker, numbers stand for reaction time: 1 stands for 10 min, 2 stands for 1 h, 3 stands for 3 h, 4 stands for 6 h, 5 stands for 24 h. 10, 100 µM, and 1 mM stand for the final concentrations of precursors. The red bracket stands for high‐molecular‐weight spider‐silk like proteins. (C) Dope solution of ∼143 mg/mL SilkC‐ADF3‐SpyT and ∼144 mg/mL SpyC‐ADF3‐SilkT in milli‐Q water. (D) Comparison of the mechanical properties of four types of fibers. Data are presented as mean ± SD (*n* ≥ 9 fibers per group). Statistical significance was determined by one‐way ANOVA. *p < 0.05, **p < 0.01, ***p < 0.001, ****p < 0.0001. (E) Representative picture of SilkC‐AQ6‐SpyT fiber under tensile test.

Two spider‐silk like protein precursors, SilkC‐AQ6‐SpyT and SpyC‐AQ6‐SilkT, were constructed. AQ6, a truncated variant of ADF3 with half‐length and enhanced solubility, which is important for spinning, was chosen as the fused silk sequence. The polymerization of spider‐silk‐like protein was achieved by simply mixing the two precursors in a ratio of 1:1 in a 50 mM sodium acetate solution, pH 5.0 (Figure 4A). Polymerization at different precursor concentrations were tested. Low precursor concentration, such as 10 µM, led to a large portion of 2x proteins formed by head‐to‐tail Catcher‐Tag cyclization reactions (Figure 4B). In contrast, when the precursor concentration was increased from 10 µM to 1 mM, the portion and size of multimeric products increased (Figure 4B). This trend is consistent with classical step‐growth polymerization theory, where intramolecular cyclization is favored at low monomer concentration and decreases with increasing precursor concentration because intermolecular ligation becomes kinetically dominant at higher monomer concentrations [61, 62].

After obtaining the ultrahigh‐molecular‐weight spider‐silk like proteins, we applied our previously established aqueous wet‐spinning method to produce spider silk fibers and evaluate their mechanical properties [60]. As discussed above, highly concentrated soluble dope solutions are essential for spinnability. Both precursor silk proteins could be concentrated to at least 100 mg/mL, sufficient for spinning in our system. However, SilkC‐fused proteins remained soluble at even higher concentrations (∼150 mg/mL), while SpyC‐fused proteins tended to aggregate at such concentrations (Figure 4C). This aggregation‐prone dope solution likely accounts for the lower spinnability and inferior mechanical performance observed in SpyC‐AQ6‐SilkT fibers (Figures 4D and S4). The spinning of ultrahigh‐molecular‐weight spider‐silk like proteins was done by mixing the two precursor dope solutions. The spinning process became less continuous and more difficult to control. Continuous spinning was achievable initially, but after ∼3 h of ligation, the viscosity of the dope solution increased, leading to only short continuous spinning. We collected fibers from the most stable spinning periods for mechanical testing. The resulting fibers showed higher stiffness but significantly reduced extensibility and toughness compared to well‐spun SilkC‐AQ6‐SpyT fibers, likely due to increased chain entanglement and restricted molecular mobility at higher molecular weights (Figures 4D and S4).

Although high‐performance fibers were not achieved through this orthogonal Catcher‐Tag pair linkage strategy, our results demonstrate the feasibility of constructing linear ultrahigh‐molecular‐weight proteins using this method. Moreover, as a solubility‐enhancing fusion tag, particularly SilkC, the Catcher domain significantly improved the solubility of recombinant spider‐silk like proteins, enabling the successful fabrication of fibers with a surprising extensibility of up to 350%, which exceeds most of the recombinant spider silk fibers reported [39, 60] (Figures 4D,E and S4). These results also demonstrated that the solubility of the silk protein is critical for a high‐quality dope solution which is a key determinant of fiber fabrication and mechanical performance.

### 2.4 Selective Affinity Recruitment Mediated by Catcher‐Tag Reaction

To investigate the potential application of orthogonal reactions between SpyC/SpyT and SilkC/SilkT pairs on the spatial organization of fused proteins, we employed recombinant spider‐silk‐like proteins that undergo liquid–liquid phase separation (LLPS) as a host to selectively recruit client proteins.

**FIGURE 5:**
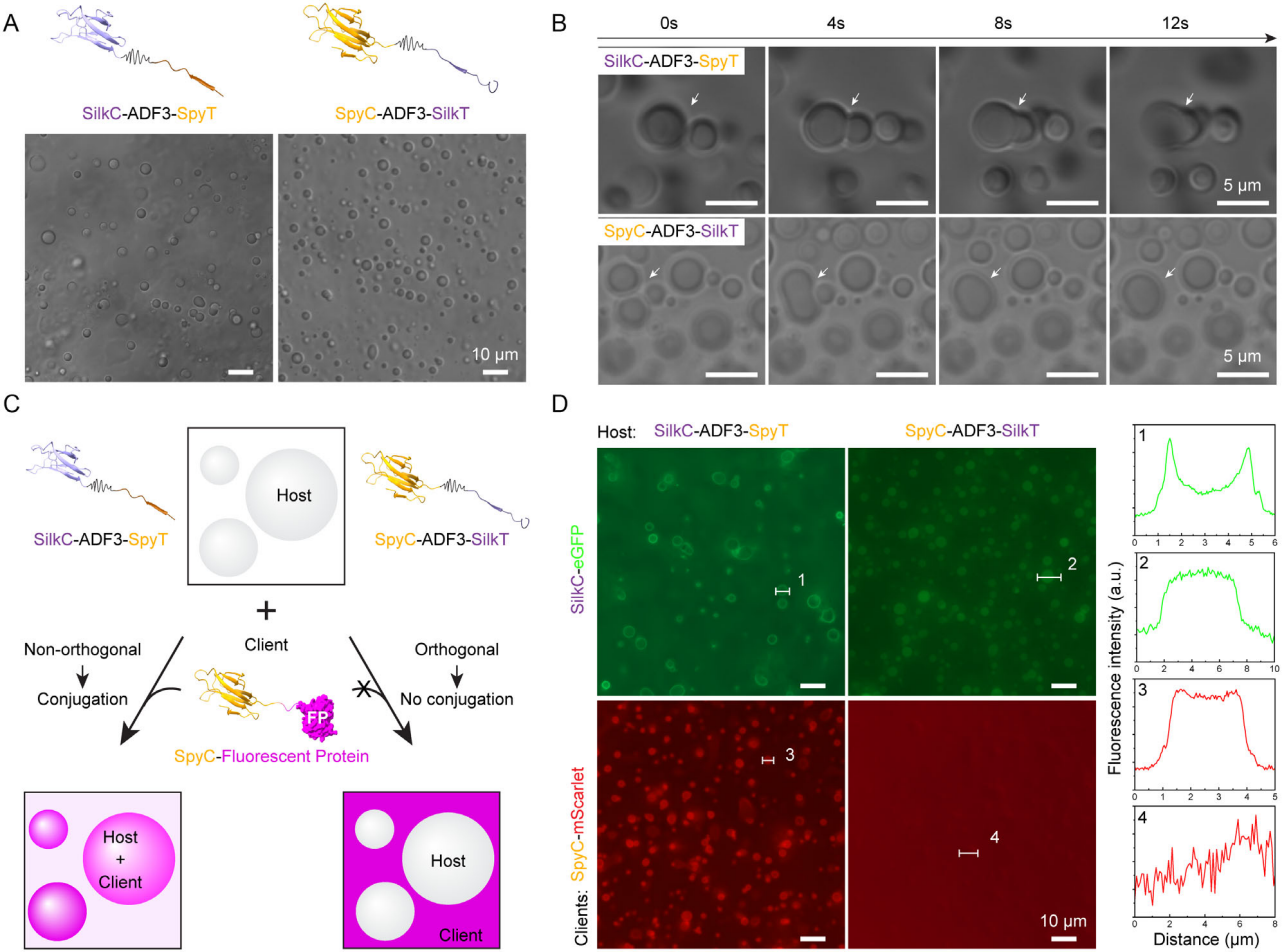
Selective recruitment through affinity between Catcher‐Tag. (A) Optical microscope image of liquid–liquid phase separation of SilkC‐ADF3‐SpyT and SpyC‐ADF3‐SilkT induced by 0.3 M phosphate buffer, pH 7.0. Scale bar is 10 µm. (B) Optical microscope images of the fusion of coacervates formed by SilkC‐ADF3‐SpyT and SpyC‐ADF3‐SilkT over time. Scale bar is 5 µm. (C) Schematic of selective recruitment through orthogonal Catcher‐Tag reaction. (D) Representative fluorescence microscopy images and quantification of client enrichment for recruitment of orthogonal Catcher fused host and client proteins. SilkC‐eGFP and SpyC‐mScarlet were recruited into different host coacervates, SpyC‐ADF3‐SilkT and SilkC‐ADF3‐SpyT. Scale bar is 10 µm.

Two constructs, SilkC‐ADF3‐SpyT and SpyC‐ADF3‐SilkT, underwent phase separation induced by phosphate buffer, forming protein‐dense droplets (Figure 5A). The rapid fusion of the droplets confirmed their liquid‐like nature (Figure 5B). Fluorescent proteins, SilkC‐eGFP and SpyC‐mScarlet, were used as client proteins to test the selective recruitment mediated by orthogonal Catcher‐Tag pairs. We hypothesized that the client proteins can only be recruited into the host coacervate when the Catcher and Tag fused with the host and client have reactivity (Figure 5C). When the SilkC‐ADF3‐SpyC served as the host, only SpyC‐mScarlet was recruited into the dense phase, while the SilkC‐eGFP remained in the surrounding solution. Conversely, when SpyC‐ADF3‐SilkT was used as the host, SilkC‐eGFP was selectively incorporated, and SpyC‐mScarlet excluded (Figure 5D). The reason for this selective recruitment is the orthogonal crosslinking reactivity between SilkC/SilkT and SpyC/SpyT pairs, which was confirmed by ligation test (Figure S5). To further confirm selective recruitment, we introduced SpyT‐eGFP into the system. When SilkC‐ADF3‐SpyT served as the host, SpyC‐mScarlet was incorporated into the condensates, while SpyT‐eGFP was excluded. In contrast, when SpyC‐ADF3‐SilkT acted as the host, SpyT‐eGFP but not SpyC‐mScarlet was recruited, demonstrating the orthogonality between the SpyC/SpyT and SilkC/SilkT pairs governs selective recruitment (Figure S6). These results highlight that orthogonal Catcher‐Tag systems enable programmable spatial organization of proteins within phase‐separated condensates, offering a versatile tool for modular assembly and compartmentalization.

**FIGURE 6:**
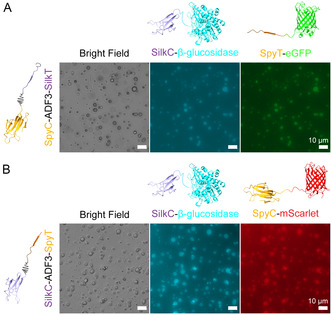
Enrichment of enzymatic reaction products in liquid–liquid phase‐separated compartments. (A) Confocal microscope images of liquid droplets formed by SpyC‐ADF3‐SilkT induced by 0.3 M phosphate buffer, pH 8.0, in the presence of SilkC‐β‐glucosidase and 2 mM 4‐methylumbelliferyl β‐D‐glucopyranoside. (B) Confocal microscope images of liquid droplets formed by SilkC‐ADF3‐SpyT induced by 0.3 M phosphate buffer, pH 8.0, in the presence of SilkC‐β‐glucosidase and 2 mM 4‐methylumbelliferyl β‐D‐glucopyranoside. Scale bar is 10 µm.

LLPS can enhance enzymatic reactions by enzymatic compartmentalization that locally concentrates enzymes and substrates [63, 64]. To further examine whether the Catcher‐Tag reaction could be used for enzyme compartmentalization, SilkC‐β‐glucosidase was employed as a model client, 4‐methylumbelliferyl‐β‐D‐glucoside (4‐MUG) as a fluorogenic substrate, and SpyC‐ADF3‐SilkT and SilkC‐ADF3‐SpyT as hosts. Covalent linkage was observed between SilkC‐β‐glucosidase and SpyC‐ADF3‐SilkT but not with SilkC‐ADF3‐SpyT (Figure S7A). Consistently, SpyC‐ADF3‐SilkT droplets showed clear enzyme enrichment, shown by the fluorescent enzymatic product, 4‐methylumbelliferone (4‐MU), and colocalization of SpyT‐eGFP (Figures 6A and S7B). However, recruitment was not strictly selective, as the SilkC‐β‐glucosidase was also enriched in SilkC‐ADF‐SpyT droplets, despite the absence of crosslinking, suggesting that nonspecific interactions between the host protein and enzyme may contribute to recruitment (Figures 6B and S7).

In summary, the Catcher‐Tag system enables selective recruitment through rationally fusing orthogonal Catcher‐Tag pairs with host and client proteins. The applicability of the system may be limited to cases in which there is no or very weak intrinsic affinity between the host and client proteins because there is a balance between intrinsic host‐client interactions and Catcher‐Tag‐mediated recruitment. When the interaction between the client and host proteins is relatively weak, the Catcher‐Tag system may primarily govern selective recruitment through orthogonal pairing. In contrast, when moderate interactions exist, they may partially interfere with or diminish the selectivity driven by the Catcher‐Tag system. By tuning the balance between intrinsic affinity and Catcher/Tag interactions, precise spatial organization of host and client proteins can still be achieved.

## 3 Conclusions

In this work, we demonstrate the versatility of the Catcher domain as a multifunctional fusion tag. By fusing the small SilkC domain with production targets, we managed to improve the soluble expression of aggregation‐prone proteins, including recombinant spider‐silk like proteins and bovine caseins, as well as enzymes. The specific structural affinity between Catcher and Tag showed the potential for protein affinity purification. Furthermore, the spontaneous covalent Catcher‐Tag reaction provided a selective and mild method for enzyme immobilization and purification. The orthogonal crosslinking activity between different Catcher‐Tag pairs further enabled the construction of linear high‐molecular‐weight spider‐silk like proteins. By combining the phase separation properties of Catcher‐fused spider‐silk like proteins and orthogonal Catcher‐Tag reactions, we achieved selective recruitment and spatial organization of client proteins. These findings highlight the Catcher domains as both a solubility‐enhancing tag and an assembly module, expanding their potential in protein engineering and in a broad range of biotechnological applications.

## 4 Experimental Section

Please see the Supporting Information for details.

## Author Contributions

**Ruxia Fan** conceptualized the work together with **A. Sesilja Aranko**, performed most of the experiments, analyzed data, and drafted the first version of the manuscript. **Xing Wan** helped with the laccase expression, collaborated with **Safoorah Khanum** for the laccase activity assay and reviewed the manuscript. **Juhani P. Jokio** helped in constructing the casein constructs. **Nea B. Möttönen** designed laccase and casein constructs. **Miia R. Mäkelä** supervised the laccase part and reviewed the manuscript. **A. Sesilja Aranko** conceptualized the work together with **Ruxia Fan**, supervised the work, and reviewed the manuscript.

## Funding

This study was supported by Academy of Finland (333238 and 366183), Novo Nordisk Fonden (NNF23OC0081564, NNF21OC0071410 and NNF21OC0067087), Emil Aaltosen Säätiö (250219 P), Jenny ja Antti Wihurin Rahasto (00230063), and Tekniikan Edistämissäätiö (10566).

## Conflicts of Interest

The authors declare no conflicts of interest.

## Supporting information

Supplementary Material

## Data Availability

The data that support the findings of this study are available from the corresponding author upon reasonable request.
