# Supplementary material for: Catcher Domains as Multifunctional Fusion Modules for Soluble Expression, Covalent Coupling, and Spatial Organization of Recombinant Proteins
Source: Small Sci. 2026 Jun 24;6(6):e70328. doi: 10.1002/smsc.70328 (PMC13292731; doi:10.1002/smsc.70328)
Supplement: Supplementary file 1 — Supplementary Material [file SMSC-6-e70328-s001.pdf]

Supporting Information

Catcher Domains as Multifunctional Fusion Modules for Soluble Expression, Covalent Coupling, and Spatial Organization of Recombinant Proteins

Ruxia Fan, Xing Wan, Safoorah Khanum, Juhani P. Jokio, Nea B. Möttönen, Miia R. Mäkelä, A. Sesilja Aranko**

Experimental Procedures

Molecular Cloning

Molecular cloning of each construct is described below.

The DNA fragments encoding ADF3, NT, GST, SUMO, CBM, SpyC, SilkC, SpyT, SilkT, bovine α_{s2}-casein, bovine β-casein, β-glucosidase, inactive SilkTag, and AQ6 were codon-optimized and synthesized by GeneArt gene synthesis (Thermo Fisher Scientific) for expression in *Escherichia coli*. The codon-optimized DNA sequence encoding SilkC-laccase in pPICZaA plasmid was synthesized by GeneArt gene synthesis (Thermo Fisher Scientific) for expression in *Pichia pastoris*.

Plasmid pSAEt56 coding for CBM-ADF3-SpyT and plasmid pSAEt266 encoding SilkC-ADF3-SpyT were constructed as reported previously[1,2]. pRFEt 20 coding for NT-ADF3-SpyT, pRFEt 19 coding for GST-ADF3-SpyT, pRFEt18 coding for SUMO-ADF3-SpyT were obtained by replacing the SilkC between *Nhe*I and *Xho*I restriction sites in plasmid pSAEt266. pRFEt22 coding for SpyC-ADF3-SilkT was constructed by replacing the backbone of pSALRSFDuet300[2] between *Nco*I and *Xho*I restriction sites with backbone of pSAEt266.

Small scale protein expression test and purification

E. coli T7 express strain (NEB) was transformed with the indicated plasmids. Cells were grown in 4 mL Luria-Bertani media supplemented with appropriate antibiotics. Protein expression was induced with 200 µM isopropyl β-D-1-thiogalactopyranoside (IPTG) at 37 °C for 3 h. The harvest cells were lysed by B-PER bacterial protein extraction reagent (Thermo Fisher Scientific). Comparable samples collected before induction, after expression, and from both pellets and supernatants fractions following lysis, and analysed by SDS-PAGE. If needed, proteins were following purified by immobilized metal affinity chromatography (IMAC) by Ni-NTA Spin Columns (QIAGEN) according to the manufacturer's protocol. The flowthrough, wash, and elution fractions were collected for SDS-PAGE analysis. For thermal stability test, equal volumes of the soluble lysate fraction were incubated at 70 °C, 80 °C, or 90 °C for 10 mins, soluble and insoluble fractions were separated by centrifugation and collected for SDS-PAGE analysis.

Protein purification mediated by SilkC and inactive SilkT

Comparable amounts of *E. coli* cells expressing SilkC-ADF3-SpyT and inactive SilkT-H6 were lysed with B-PER bacterial protein extraction reagent (Thermo Fisher Scientific). The soluble fraction after lysis were collected and adjusted to pH 5.0 with 20 mM phosphate buffer (PB). 50 µL inactive SilkT-H6 supernatant was loaded onto Ni-NTA Spin Column (QIAGEN). 150 µL SilkC-ADF3-SpyT supernatant was then added to the same column and incubated overnight. The column was washed with 200 µL of 20 mM PB buffer, pH 5.0. Elution was performed sequentially with 300 µL 300 mM NaCl, 20 mM Tris-HCl buffer, pH 7.4 (Elution buffer E), to elute SilkC-ADF3-SpyT, followed by 100 µL 250 mM imidazole, 300 mM NaCl, 20 mM Tris-HCl buffer, pH 7.4 (Elution buffer E'), to elute inactive SilkT-H6. All the flowthrough, wash, and elution fractions were collected for SDS-PAGE analysis.

Large scale protein expression and purification

E. coli T7 express strain (NEB) was transformed with the indicated plasmids. Cells were cultured in 0.5 L EnPresso B500 medium (EnPresso) supplemented with kanamycin (50 µg/mL) according to the manufacturer's protocol. Cultures were harvested by centrifugation at 10,000 ×g for 15 mins at 4 °C. Pellets were resuspended into Lysis Buffer (50 mM Tris–HCl, pH 7.4, containing 3 mM $MgCl_2$, 100 mM NaCl, 1 mg/mL lysozyme (EC 3.2.1.17, Merck), 10 µg/mL DNase I (EC 3.1.21.1, Merck), and one protease inhibitor tablet per 50 mL buffer (Thermo Fisher Scientific)). The resuspended cells were incubated for 1 h at 37 °C and 150 rpm, followed by sonication using a Qsonica 500 sonicator (Qsonica LLC) for 4 × 2 min cycles at 30% amplitude. The soluble fractions were obtained by centrifugation at 10,000 × g for 15 min at 4 °C. The soluble fractions were purified by IMAC using 5 mL HisTrap HP column (Cytiva). Elution fractions containing the target proteins were pooled and buffer-exchanged into Milli-Q water using Econo-Pac 10DG columns (Bio-Rad). Proteins were concentrated with Vivaspin centrifugal concentrators (Sartorius) with a 10 kDa molecular weight cutoff (MWCO) or stored in −80 °C until further use.

Enzyme immobilization of SilkC-β-Glucosidase

Immobilization of β-glucosidase was done by two step coupling; first step was coupling SilkTag to Fe_3O_4 nanoparticles decorated by NH_2 (Sigma Aldrich) by using 1-Ethyl-3-[3-dimethylaminopropyl] carbodiimide hydrochloride (EDC, Sigma Aldrich) and N-

hydroxysulfosuccinimide (Sulfo-NHS, Sigma Aldrich). EDC and Sulfo-NHS were freshly made with final concentration of 200 mM. Synthetic SilkTag peptide was dissolved in 50 mM MES buffer (2-morpholinoethane sulfonic acid, pH 5.0) in 400 µM final concentration. EDC and NHS were added into SilkTag solution with molar ratio in EDC: NHS: SilkTag = 10: 25: 1.0. After gently mixing and 15 mins reaction, the pH of the solution rapidly shifted to pH 7.0~7.5 by adding several drops of 1 M HEPES buffer, pH 8.0. pH of the solution was monitored by pH meter (Mettler Toledo). Fe_3O_4@NH_2 was added into the SilkTag solution and incubated at room temperature for 2 h to couple SilkTag to Fe_3O_4 nanoparticles. Nanoparticles were collected by magnet and washed twice by 50 mM MES buffer, pH 5.0. Second step was immobilizing SilkC-β-glucosidase on Fe_3O_4@NH_2-SilkTag nanoparticles made in first step by Catcher-Tag click reaction. Fe_3O_4@NH_2-SilkTag nanoparticles were resuspended by excess SilkC-β-glucosidase solution with incubation at 16 °C for 48 h. After incubation, nanoparticles were collected by magnet and washed twice by 50 mM MES buffer, pH 5.0. The immobilization was monitored by SDS-PAGE, samples were taken from SilkC-β-glucosidase stock solution and separated supernatant after immobilization.

Production of SilkC-laccase in *P. pastoris*

The pPICZaA plasmid carrying SilkC-laccase was transformed into *E. coli* DH5a cells, amplified and linearized at *Pme*I site before transformation (MicroPulser™ electroporator, Bio-Rad) into electrocompetent *P. pastoris* X-33 cells. [3] Transformants were screened for secretion of active SilkC-laccase on 2,2′-azino-bis(3-ethylbenzothiazoline-6-sulfonic acid) (ABTS, Sigma Aldrich) agar plates, [4] and transformants showing a green halo around the colonies were selected for production in shake flasks as described previously [5].The supernatants from the recombinant *P. pastoris* cultures were collected by centrifugation (Sorvall LYNX 4000 centrifuge, Thermo Scientific), and filtrated with Whatman glass microfiber filters (Merck). The cell free laccase extracts were stored at 4 °C.

Enzyme activity and reuse analysis

The activity of free and immobilized β-glucosidase was evaluated using p-nitrophenyl-β-D-glucopyranoside (pNPG) as the substrate. For temperature dependent activity, aliquots of free or immobilized enzyme were incubated with pNPG in 50 mM McIlvaine buffer, pH 5.0, at different temperature 25, 35, 45, 55, and 65 °C for 10 min. For activity test under different pH, enzyme activity was assayed at 45 °C in 50 Mm McIlvaine buffers at pH 3.0, 4.0, 5.0, 6.0, 7.0, and 8.0 with pNPG. Reactions were stopped by adding 1 M Na_2CO_3, and the absorbance of the released p-nitrophenol was measured at 405 nm. Activity was expressed relative to the maximal observed activity. Reusability test of immobilized SilkC-β-glucosidase was performed in 50 mM McIlvaine buffer, pH 5.0, at 45 °C with pNPG. After each reaction cycle, immobilized enzyme was collected using a magnetic stand, washed with reaction buffer, and applied to fresh pNPG substrate. Residual activity after each cycle was measured as described above to evaluate reusability. SilkC-β-glucosidase activity was evaluated using 4-methylumbelliferyl-β-D-glucoside (4-MUG) as a fluorogenic substrate. Reactions were performed at room temperature, 22 ± 2 °C, in 50 mM McIlvaine buffer, pH 7.0 for 10 mins and stopped by adding 1M Na_2CO_3. Fluorescence of the released 4-methylumbelliferone was monitored with excitation at 365 nm, and emission was recorded from 420 to 700 nm.

Laccase activity was determined by following the oxidation of ABTS at 420 nm [6] in McIlvaine buffer, pH 4.0, with Cytation3 microplate reader (BioTek) using triplicate samples.

Polymerization of spider-silk like proteins

Polymerization was conducted by mixing two precursor spider-silk like proteins. Purified and concentrated precursors SilkC-AQ6-SpyT and SpyC-AQ6-SilkT were mixed in ratio of 1:1. Ligation reactions were performed at 37 °C for 24 h with precursor concentrations of 10, 100, or 1000 µM. Concentrations of the precursors were determined from Coomassie blue-stained SDS-PAGE gels using Image Lab 6.0.1 software (Bio-Rad), based on band intensity comparisons to an IMAC-purified reference sample of which the concentration had been determined by Nanodrop 2000 Spectrophotometers (Thermo Fisher Scientific). Reactions were terminated by adding SDS–PAGE loading buffer and heating at 95 °C for 5 min.

Production and characterization of fibers

Wet-spinning and tensile test were performed as previously described. Briefly, spinning dopes were prepared from concentrated protein solutions at 10–15% (w/v). For high-molecular-weight protein fibers, two precursor dopes were mixed at a 1:1 ratio prior to spinning. The dope solution was extruded into a coagulation bath containing 4 M ammonium acetate (pH 5.0) at a flow rate of 0.1 $\mu L \cdot s^{-1}$. Fibers were collected in air on rectangle frames attached to a roller at constant speed of 30 cm s^{-1}. All spinning processes were carried out

under room temperature, 22 ± 2 °C, and the fibers were stored in a humidity chamber at 20% relative humidity (RH). For tensile testing, fibers were mounted on paper frames and tested by Universal Tester Instron 5944 equipped with a 5 N load cell. Fiber diameters were measured by Axio Vert.A1 inverted optical microscope with 20x objective lens. To simplify the calculation, we assumed that the cross-sections of fibers were circular. The diameter of each single fiber was determined as the average from nine measurements taken from three micrographs at different fiber positions. Tensile tests were conducted at a strain rate of 2 mm·min^{-1} under 43 ± 2% RH. At least 9 fibers were tested for each sample. The strain, fracture strength, Young's modulus, and toughness modulus were determined following the procedure described in ref[7]. In brief, the true engineering stress and strain were calculated. The Young's modulus was obtained from the slope of the initial linear region of the stress-strain curves. The Toughness was calculated as the area under the stress-strain curve.

Fluorescent microscope

Imaging was conducted using an Axio Observer Z1 microscope (Carl Zeiss) equipped with Andor iXon Ultra 888 cameras. The GFP fluorescence was excited at 470 nm, and emission was collected between 515–535 nm. mScarlet fluorescence was excited at 590 nm, and emission was collected between 610–635 nm. 4-methylumbelliferone fluorescence was excited at 420 nm, and emission was collected between 461–485 nm. Phase separation samples were prepared by mixing 10 mg/ml SilkC-ADF3-SpyT or SpyC-ADF3-SilkT protein solution with 0.5 M potassium phosphate buffer, pH 7.0 in ratio of 1:1. Recruitment samples were prepared by adding the client protein with the ratio of Catcher from host or client:Tag from host or client equal to 1:1 and incubate for 10 mins before imaging. Recruitment of SilkC-β-glucosidase was performed in 0.5 M potassium phosphate buffer, pH 8.0 to induce the fluorescence of catalytic product, 4-methylumbelliferone, 4-MU. Protein samples were placed on a glass slide and observed.

Table S1. Amino-acid sequences of constructs used in this article.

Name	Amino acid sequences
NT-ADF3-SpyT<br>(62.922 kDa)	MGSGNSHTTPWTNPGLAENFMNSFMQGLSSMPGFTASQLDDMSTIAQSMVQSIQSLAA<br>QGRTSPNKLQALNMAFASSMAEIAASEEGGGSLSTKTSSIASAMSNAFLQTTGVVNQPFI<br>NEITQLVSMFAQAGMNDVSASASASAGASAAASAGAGAGAGPGQQGPGQQGPGQQGP<br>YGPGASAAAAAAGGYGPGSGQQGPSQQGPGQQGPGGQGPYGPGASAAAAAAGGYGP<br>GSGQQGPGGQGPYGPGSSAAAAAAGGNGPGSGQQGAGQQGPGQQGPGGSAAAAAAG<br>GYGPGSGQQGPGQQGPGGQGPYGPGASAAAAAAGGYGPGSGQGPGQQGPGGQGPYG<br>PGASAAAAAAGGYGPGSGQQGPGQQGPGQQGPGGQGPYGPGASAAAAAAGGYGPGY<br>GQQGPGQQGPGGQGPYGPGASAASAASGGYGPGSGQQGPGQQGPGGQGPYGPGASAA<br>AAAAGGYGPGSGQQGPGQQGPGQQGPGQQGPGGQGPYGPGASAAAAAAGGYGPGSG<br>QQGPGQQGPGQQGPGQQGPGQQGPGQQGPGQQGPGQQGPGQQGPGGQGAYGPGASA<br>AAGAAGGYGPGSGQQGPGQQGPGQQGPGQQGPGQQGPGQQGPGQQGPGQQGPYGPG<br>ASAAAAAAGGYGPGSGQQGPGQQGPGQQGPGGQASASASAAASAASTVANSSSAHIV<br>MVDAYKPTKLEHHHHHH
GST-ADF3-SpyT<br>(75.050 kDa)	MGSPILGYWKIKGLVQPTRLLLEYLEEKYEEHLYERDEGDKWRNKKFELGLEFPNLPYY<br>IDGDVKLTQSMAIIRYIADKHNMLGGCPKERAEISMLEGAVLDIRYGVSRIAYSKDFETLK<br>VDFLSKLPEMLKMFEDRLCHKTYLNGDHVTHPDFMLYDALDVVLYMDPMCLDAFPKL<br>VCFKKRIEAIPQIDKYLKSSKYIAWPLQGWQATFGGGDHPPKSDLVPRGSASASASAGAS<br>AAASAGAGAGAGPGQQGPGQQGPGQQGPYGPGASAAAAAAGGYGPGSGQQGPSQQG<br>PGQQGPGGQGPYGPGASAAAAAAGGYGPGSGQQGPGGQGPYGPGSSAAAAAAGGNG<br>PGSGQQGAGQQGPGQQGPGGSAAAAAAGGYGPGSGQQGPGQQGPGGQGPYGPGASA<br>AAAAAGGYGPGSGQGPGQQGPGGQGPYGPGASAAAAAAGGYGPGSGQQGPGQQGPG<br>QQGPGGQGPYGPGASAAAAAAGGYGPGYGQQGPGQQGPGGQGPYGPGASAASAASG<br>GYGPGSGQQGPGQQGPGGQGPYGPGASAAAAAAGGYGPGSGQQGPGQQGPGQQGPG<br>QQGPGGQGPYGPGASAAAAAAGGYGPGSGQQGPGQQGPGQQGPGQQGPGQQGPGQQ<br>GPGQQGPGQQGPGQQGPGGQGAYGPGASAAAGAAGGYGPGSGQQGPGQQGPGQQGP<br>GQQGPGQQGPGQQGPGQQGPGQQGPYGPGASAAAAAAGGYGPGSGQQGPGQQGPGQ<br>QGPGGQASASASAAASAASTVANSSSAHIVMVDAYKPTKLEHHHHHH
SUMO-ADF3-SpyT<br>(60.088 kDa)	MGSDSEVNQEAKPEVKPEVKPETHINLKVSDGSSEIFFKIKKTTPLRRLMEAFAKRQGKE<br>MDSLRFLYDGIRIQADQTPEDLDMEDNDIIEAHREQIGGSASASASAGASAAASAGAGA<br>GAGPGQQGPGQQGPGQQGPYGPGASAAAAAAGGYGPGSGQQGPSQQGPGQQGPGGQ<br>GPYGPGASAAAAAAGGYGPGSGQQGPGGQGPYGPGSSAAAAAAGGNGPGSGQQGAG<br>QQGPGQQGPGGSAAAAAAGGYGPGSGQQGPGQQGPGGQGPYGPGASAAAAAAGGYG<br>PGSGQGPGQQGPGGQGPYGPGASAAAAAAGGYGPGSGQQGPGQQGPGQQGPGGQGP<br>YGPGASAAAAAAGGYGPGYGQQGPGQQGPGGQGPYGPGASAASAASGGYGPGSGQQ<br>GPGQQGPGGQGPYGPGASAAAAAAGGYGPGSGQQGPGQQGPGQQGPGQQGPGGQGP<br>YGPGASAAAAAAGGYGPGSGQQGPGQQGPGQQGPGQQGPGQQGPGQQGPGQQGPGQ<br>QGPGQQGPGGQGAYGPGASAAAGAAGGYGPGSGQQGPGQQGPGQQGPGQQGPGQQG<br>PGQQGPGQQGPGQQGPYGPGASAAAAAAGGYGPGSGQQGPGQQGPGQQGPGGQASA<br>SASAAASAASTVANSSSAHIVMVDAYKPTKLEHHHHHH
CBM-ADF3-SpyT<br>(66.174 kDa)	MGNLKVEFYNSNPSDTTNSINPQFKVTNTGSSAIDLSKLTLRYYYTVDGQKDQTFWCDH<br>AAIIGSNGSYNGITSNVKGTFVKMSSSTNNADTYLEISFTGGTLEPGAHVQIQGRFAKND<br>WSNYTQSNDYSFKSASQFVEWDQVTAYLNGVLVWGKEPSASASASAGASAAASAGAG<br>AGAGPGQQGPGQQGPGQQGPYGPGASAAAAAAGGYGPGSGQQGPSQQGPGQQGPGG<br>QGPYGPGASAAAAAAGGYGPGSGQQGPGGQGPYGPGSSAAAAAAGGNGPGSGQQGA<br>GQQGPGQQGPGGSAAAAAAGGYGPGSGQQGPGQQGPGGQGPYGPGASAAAAAAGGY<br>GPGSGQGPGQQGPGGQGPYGPGASAAAAAAGGYGPGSGQQGPGQQGPGQQGPGGQG<br>PYGPGASAAAAAAGGYGPGYGQQGPGQQGPGGQGPYGPGASAASAASGGYGPGSGQ<br>QGPGQQGPGGQGPYGPGASAAAAAAGGYGPGSGQQGPGQQGPGQQGPGQQGPGGQG<br>PYGPGASAAAAAAGGYGPGSGQQGPGQQGPGQQGPGQQGPGQQGPGQQGPGQQGPG<br>QQGPGQQGPGGQGAYGPGASAAAGAAGGYGPGSGQQGPGQQGPGQQGPGQQGPGQQ<br>GPGQQGPGQQGPGQQGPYGPGASAAAAAAGGYGPGSGQQGPGQQGPGQQGPGGQAS<br>ASASAAASAASTVANSSSAHIVMVDAYKPTKLEHHHHHH
SpyC-ADF3-SilkT<br>(62.309 kDa)	MGAMVTTLSGLSGEQGPSGDMTTEEDSATHIKFSKRDEDGRELAGATMELRDSSGKTIS<br>TWISDGHVKDFYLYPGKYTFVETAAPDGYEVATAITFTVNEQGQVTVNGEATKGDAHTG<br>SPSASASASAGASAAASAGAGAGAGPGQQGPGQQGPGQQGPYGPGASAAAAAAGGYG<br>PGSGQQGPSQQGPGQQGPGGQGPYGPGASAAAAAAGGYGPGSGQQGPGGQGPYGPGS<br>SAAAAAAGGNGPGSGQQGAGQQGPGQQGPGGSAAAAAAGGYGPGSGQQGPGQQGPG<br>GQGPYGPGASAAAAAAGGYGPGSGQGPGQQGPGGQGPYGPGASAAAAAAGGYGPGS

	GQQGPGQQGPGQQGPGGQGPYGPGASAAAAAAGGYGPGYGQQGPGQQGPGGQGPYG<br>PGASAASAASGGYGPGSGQQGPGQQGPGGQGPYGPGASAAAAAAGGYGPGSGQQGPG<br>QQGPGQQGPGQQGPGGQGPYGPGASAAAAAAGGYGPGSGQQGPGQQGPGQQGPGQQ<br>GPGQQGPGQQGPGQQGPGQQGPGQQGPGGQGAYGPGASAAAGAAGGYGPGSGQQGP<br>GQQGPGQQGPGQQGPGQQGPGQQGPGQQGPGQQGPYGPGASAAAAAAGGYGPGSGQ<br>QGPGQQGPGQQGPGGQASASASAAASAASTVANSSSGIKPEVAFQVSQDDVKQPVVPT<br>GTHHHHHH
SilkC-ADF3-SpyT<br>(57.446 kDa)	MGTYTIELTKHDAATKAVLAGAVYELQDSTGKVIQTGLTTDSQGQLIVKNLRAGDYQFV<br>ETKAPLGYELNTTPVKFTLGSPSASASASAGASAAASAGAGAGAGPGQQGPGQQGPGQ<br>QGPYGPGASAAAAAAGGYGPGSGQQGPSQQGPGQQGPGGQGPYGPGASAAAAAAGG<br>YGPGSGQQGPGGQGPYGPGSSAAAAAAGGNGPGSGQQGAGQQGPGQQGPGGSAAAA<br>AAGGYGPGSGQQGPGQQGPGGQGPYGPGASAAAAAAGGYGPGSGQGPGQQGPGGQG<br>PYGPGASAAAAAAGGYGPGSGQQGPGQQGPGQQGPGGQGPYGPGASAAAAAAGGYG<br>PGYGQQGPGQQGPGGQGPYGPGASAASAASGGYGPGSGQQGPGQQGPGGQGPYGPGA<br>SAAAAAAGGYGPGSGQQGPGQQGPGQQGPGQQGPGGQGPYGPGASAAAAAAGGYGP<br>GSGQQGPGQQGPGQQGPGQQGPGQQGPGQQGPGQQGPGQQGPGQQGPGGQGAYGPG<br>ASAAAGAAGGYGPGSGQQGPGQQGPGQQGPGQQGPGQQGPGQQGPGQQGPGQQGPY<br>GPGASAAAAAAGGYGPGSGQQGPGQQGPGQQGPGGQASASASAAASAASTVANSSSA<br>HIVMVDAYKPTKLEHHHHHH
α_{s2}-Casein<br>(18.857 kDa)	MAGSEFGKNTMEHVSSSEESIISQETYKQEKNMAINPSKENLCSTFCKEVVRNANEEEYS<br>IGSSSEESAEVATEEVKITVDDKHYQKALNEINQFYQKFPQYLQYLYQGPIVLNPWDQVK<br>RNAVPITPTLNREQLSTSEENSKKTVDMESTEVFTKGGGHHHHHH
SpyC-α_{s2}-Casein<br>(31.311 kDa)	MAGSGMVTTLSGLSGEQGPSGDMTTEEDSATHIKFSKRDEDGRELAGATMELRDSSGKT<br>ISTWISDGHVKDFYLYPGKYTFVETAAPDGYEVATAITFTVNEQGQVTVNGEATKGDAH<br>TGGSEFGKNTMEHVSSSEESIISQETYKQEKNMAINPSKENLCSTFCKEVVRNANEEEYSI<br>GSSSEESAEVATEEVKITVDDKHYQKALNEINQFYQKFPQYLQYLYQGPIVLNPWDQVK<br>RNAVPITPTLNREQLSTSEENSKKTVDMESTEVFTKGGGHHHHHH
SilkC-α_{s2}-Casein<br>(27.476 kDa)	MAGSMTYTIELTKHDAATKAVLAGAVYELQDSTGKVIQTGLTTDSQGQLIVKNLRAGDY<br>QFVETKAPLGYELNTTPVKFTLGGGSEFGKNTMEHVSSSEESIISQETYKQEKNMAINPS<br>KENLCSTFCKEVVRNANEEEYSIGSSSEESAEVATEEVKITVDDKHYQKALNEINQFYQK<br>FPQYLQYLYQGPIVLNPWDQVKRNAVPITPTLNREQLSTSEENSKKTVDMESTEVFTKG<br>GGHHHHHH
β-Casein<br>(25.257 kDa)	MAGSEFGRELEELNVPGEIVESLSSSEESITRINKKIEKFQSEEQQQTEDELQDKIHPFAQT<br>QSLVYPFPGPIPNSLPQNIPPLTQTPVVVPPFLQPEVMGVSKVKEAMAPKHKEMPFPKYP<br>VEPFTESQSLTLTDVENLHLPLPLLQSWMHQPHQPLPPTVMFPPQSVLSLSQSKVLPVPQ<br>KAVPYPQRDMPIQAFLLYQEPVLGPVRGPFPIIVGGGHHHHHH
SpyC-β-Casein<br>(37.712 kDa)	MAGSGMVTTLSGLSGEQGPSGDMTTEEDSATHIKFSKRDEDGRELAGATMELRDSSGKT<br>ISTWISDGHVKDFYLYPGKYTFVETAAPDGYEVATAITFTVNEQGQVTVNGEATKGDAH<br>TGGSEFGRELEELNVPGEIVESLSSSEESITRINKKIEKFQSEEQQQTEDELQDKIHPFAQT<br>QSLVYPFPGPIPNSLPQNIPPLTQTPVVVPPFLQPEVMGVSKVKEAMAPKHKEMPFPKYP<br>VEPFTESQSLTLTDVENLHLPLPLLQSWMHQPHQPLPPTVMFPPQSVLSLSQSKVLPVPQ<br>KAVPYPQRDMPIQAFLLYQEPVLGPVRGPFPIIVGGGHHHHHH
SilkC-β-Casein<br>(33.876 kDa)	MAGSMTYTIELTKHDAATKAVLAGAVYELQDSTGKVIQTGLTTDSQGQLIVKNLRAGDY<br>QFVETKAPLGYELNTTPVKFTLGGGSEFGRELEELNVPGEIVESLSSSEESITRINKKIEKF<br>QSEEQQQTEDELQDKIHPFAQTQSLVYPFPGPIPNSLPQNIPPLTQTPVVVPPFLQPEVMG<br>VSKVKEAMAPKHKEMPFPKYPVEPFTESQSLTLTDVENLHLPLPLLQSWMHQPHQPLPP<br>TVMFPPQSVLSLSQSKVLPVPQKAVPYPQRDMPIQAFLLYQEPVLGPVRGPFPIIVGGGH<br>HHHHH
β-Glucosidase<br>(61.356 kDa)	MGHHHHHHSSGLVPRGSHMAGTPSKPSEPIGPVFTKLKPWQIPKRDWFSKDFLFGASTS<br>AYQIEGAWNEDGKGPSTWDHFCHTYPERISDGTNGDVAANSYHMYEEDVKALKDMGM<br>KVYRFSISWSRILPNGTGKPNQKGIDYYNNLINSLIRHGIVPYVTIWHWDTPQALEDKYG<br>GFLDKQIVNDYKYFAELCFQSFGDRVKNWFTFNEPHTYCCFSYGEGIHAPGRCSPGLDC<br>AVPEGDSLREPYTAGHHILLAHAEAVELFKAHYNKHGDSKIGMAFDVMGYEPYQDSFL<br>DDQARERSIDYNMGWFLEPVVRGDYPFSMRSLIGDRLPMFTKEEQEKLASSCDIMGLNY<br>YTSRFSKHVDISSDYTPTLNTDDAYASSETTGSDGNEIGPITGTYWIYMYPKGLTDLLLIM<br>KEKYGNPPIFITENGIADVEGDPEMPDPLDDWKRLDYLQRHISAVKDAIDQGADVRGHF<br>TWGLIDNFEWGSGYSSRFGLVYIDKEDGNKRKLKKSAKWFAKFNSVPKTLLKTTNNNA<br>TVTASVSVEFG
SilkC-β-Glucosidase<br>(68.756 kDa)	MGHHHHHHSGTYTIELTKHDAATKAVLAGAVYELQDSTGKVIQTGLTTDSQGQLIVKNL<br>RAGDYQFVETKAPLGYELNTTPVKFTLGSGHMAGTPSKPSEPIGPVFTKLKPWQIPKRD<br>WFSKDFLFGASTSAYQIEGAWNEDGKGPSTWDHFCHTYPERISDGTNGDVAANSYHMY<br>EEDVKALKDMGMKVYRFSISWSRILPNGTGKPNQKGIDYYNNLINSLIRHGIVPYVTIW

	HWDTPQALEDKYGGFLDKQIVNDYKYFAELCFQSFGDRVKNWFTFNEPHTYCCFSYGE<br>GIHAPGRCSPGLDCAVPEGDSLREPYTAGHHILLAHAEAVELFKAHYNKHGDSKIGMAF<br>DVMGYEPYQDSFLDDQARERSIDYNMGWFLEPVVRGDYPFSMRSLIGDRLPMFTKEEQ<br>EKLASSCDIMGLNYYTSRFSKHVDISSDYTPTLNTDDAYASSETTGSDGNEIGPITGTYWI<br>YMYPKGLTDLLLIMKEKYGNPPIFITENGIADVEGDPEMPDPLDDWKRLDYLQRHISAV<br>KDAIDQGADVRGHFTWGLIDNFEWGSGYSSRFGLVYIDKEDGNKRKLKKSAKWFAKFN<br>SVPKTLLKTTNNNATVTASVSV
SilkC-Laccase<br>(74.120 kDa)	MRFPSIFTAVLFAASSALAAPVNTTTEDETAQIPAEAVIGYSDLEGDFDVAVLPFSNSTNNG<br>LLFINTTIASIAAKEEGVSLEKREAEAEFMTYTIELTKHDAATKAVLAGAVYELQDSTGK<br>VIQTGLTTDSQGQLIVKNLRAGDYQFVQTKAPLGYELNTTPVKFTLGASASASAAASAA<br>LVPRGSSSAIGPVTDLRITNKNVSPDGVSRPAVLAGGTLPGPTIKGNKGDHFKINVIDELT<br>NPDMFKSTSIHWHGLFQKGTNWADGPAFVTQCPITTGNSFLYDFRVPDQAGTFWYHSHL<br>SVQYCDGLRGPMVIYDPHDPHKDLYDVDDDSTIITLADWYHTLARQITGPARSDATLIN<br>GLGRSPTGPPDAELAVITVQRNKRYRFRLVSLSCDPNFIFSIDNHTMTVIEVDGENTQSLT<br>VDSIQIFAGQRYSFVLHANQPENNYWIRAQPNTGNTTFIGGLNSAILRYVGAPKKEPVTV<br>QQPSKTPLKEIDLRPLKYTPVPGKPYPGGADIVKNLALSFNGGKFFINNQSFVPPTVPVLL<br>QILSGHHDASELLPPGGVIGLERNKVVEITLPGGVIGSPHPFHLHGHSFWVVRSAGSDKY<br>NFNDPVIRDVVNIGGNSDDLVTIRFVTDNPGPWFLHCHIDFHLEAGFAIVFAEGINETAAA<br>NPVPHAWYDLCPKYDALAPGDQ
Inactive SilkT-H6<br>(21.081 kDa)	MGIKPEVAFQVSQDAVKQPVVPTANSSSNLKVEFYNSNPSDTTNSINPQFKVTNTGSSAI<br>DLSKLTLRYYYTVDGQKDQTFWCDHAAIIGSNGSYNGITSNVKGTFVKMSSSTNNADT<br>YLEISFTGGTLEPGAHVQIQGRFAKNDWSNYTQSNDYSFKSASQFVEWDQVTAYLNGVL<br>VWGKELEHHHHHH
eGFP-SilkC<br>(37.649 kDa)	MGMGSSHHHHHHSSGLVPRGSHVSKGEELFTGVVPILVELDGDVNGHKFSVSGEGEGD<br>ATYGKLTLKFICTTGKLPVPWPTLVTTLTYGVQCFSRYPDHMKQHDFFKSAMPEGYVQE<br>RTIFFKDDGNYKTRAEVKFEGDTLVNRIELKGIDFKEDGNILGHKLEYNYNSHNVYIMA<br>DKQKNGIKVNFKIRHNIEDGSVQLADHYQQNTPIGDGPVLLPDNHYLSTQSALSKDPNE<br>KRDHMVLLEFVTAAGITLGMDELYKGGSTYTIELTKHDAATKAVLAGAVYELQDSTGK<br>VIQTGLTTDSQGQLIVKNLRAGDYQFVETKAPLGYELNTTPVKFTLG
mScarlet-SpyC<br>(41.241 kDa)	MGMGSSHHHHHHSSGLVPRGSHMVSKGEAVIKEFMRFKVHMEGSMNGHEFEIEGEGEG<br>RPYEGTQTAKLKVTKGGPLPFSWDILSPQFMYGSRAFIKHPADIPDYYKQSFPEGFKWER<br>VMNFEDGGAVTVTQDTSLEDGTLIYKVKLRGTNFPPDGPVMQKKTMGWEASTERLYPE<br>DGVLKGDIKMALRLKDGGRYLADFKTTYKAKKPVQMPGAYNVDRKLDITSHNEDYTV<br>VEQYERSEGRHSTGGMDELYKGGSGAMVTTLSGLSGEQGPSGDMTTEEDSATHIKFSKR<br>DEDGRELAGATMELRDSSGKTISTWISDGHVKDFYLYPGKYTFVETAAPDGYEVATAITF<br>TVNEQGQVTVNGEATKGDAHT
eGFP-SpyTag<br>(30.746 kDa)	MHHHHHHSSGVSKGEELFTGVVPILVELDGDVNGHKFSVSGEGEGDATYGKLTLKFICT<br>TGKLPVPWPTLVTTLTYGVQCFSRYPDHMKQHDFFKSAMPEGYVQERTIFFKDDGNYK<br>TRAEVKFEGDTLVNRIELKGIDFKEDGNILGHKLEYNYNSHNVYIMADKQKNGIKVNFK<br>IRHNIEDGSVQLADHYQQNTPIGDGPVLLPDNHYLSTQSALSKDPNEKRDHMVLLEFVT<br>AAGITLGMDELYKSASASASAGAAHIVMVDAYKPTK
SpyC-AQ6-SilkT<br>(38.957 kDa)	MGAMVTTLSGLSGEQGPSGDMTTEEDSATHIKFSKRDEDGRELAGATMELRDSSGKTIS<br>TWISDGHVKDFYLYPGKYTFVETAAPDGYEVATAITFTVNEQGQVTVNGEATKGDAHTG<br>SGPYGPGASAAAAAAGGYGPGSGQQGPGQQGPGQQGPQQGPGGQGPYGPGASAAAA<br>AAGGYGPGSGQQGPGQQGPGQQGPQQGPGGQGPYGPGASAAAAAAGGYGPGSGQQG<br>PGQQGPGQQGPQQGPGGQGPYGPGASAAAAAAGGYGPGSGQQGPGQQGPGQQGPQQ<br>GPGGQGPYGPGASAAAAAAGGYGPGSGQQGPGQQGPGQQGPQQGPGGQGPYGPGAS<br>AAAAAAGGYGPGSGQQGPGQQGPGQQGPQQGPGGQRNSSSGIKPEVAFQVSQDDVKQ<br>PVVPTGTHHHHHH
SilkC-AQ6-SpyT<br>(34.094 kDa)	MGTYTIELTKHDAATKAVLAGAVYELQDSTGKVIQTGLTTDSQGQLIVKNLRAGDYQFV<br>ETKAPLGYELNTTPVKFTLGSGPYGPGASAAAAAAGGYGPGSGQQGPGQQGPGQQGPQ<br>QGPGGQGPYGPGASAAAAAAGGYGPGSGQQGPGQQGPGQQGPQQGPGGQGPYGPGA<br>SAAAAAAGGYGPGSGQQGPGQQGPGQQGPQQGPGGQGPYGPGASAAAAAAGGYGPG<br>SGQQGPGQQGPGQQGPQQGPGGQGPYGPGASAAAAAAGGYGPGSGQQGPGQQGPGQ<br>QGPQQGPGGQGPYGPGASAAAAAAGGYGPGSGQQGPGQQGPGQQGPQQGPGGQRNSS<br>SAHIVMVDAYKPTKLEHHHHHH

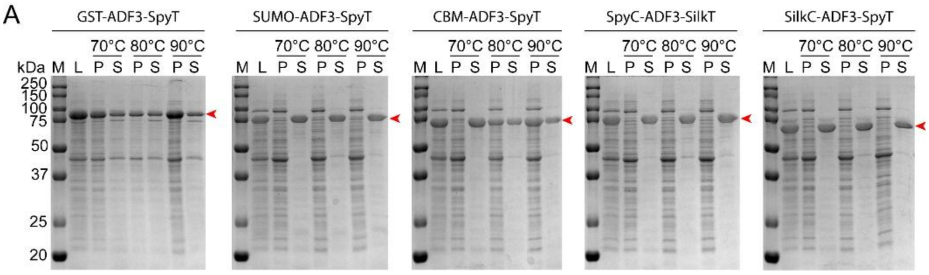

Figure S1. Thermal stability of recombinant spider-silk like proteins fused with different fusion tags. M stands for marker, L stands for supernatant from cell lysate, P stands for pellet after incubation at a given temperature, and S stands for supernatant after incubation at a given temperature. Theoretical molecular weights of five recombinant spider silk-like proteins: GST-ADF3-SpyT is 75.1 kDa, SUMO-ADF3-SpyT is 60.1 kDa, CBM-ADF3-SpyT is 66.2 kDa, SpyC-ADF3-SilkT is 62.3 kDa, and SilkC-ADF3-SpyT is 57.4 kDa.

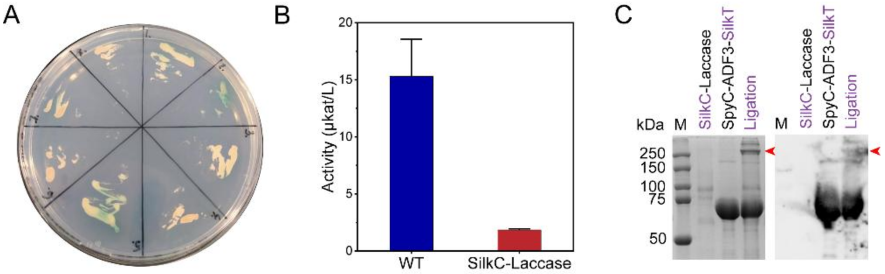

Figure S2. Laccase expression and activity test. A) Laccase activity screening on an ABTS plate of *P. pastoris* transformants producing SilkC-Laccase. B) Activity of wide-type laccase (WT) and SilkC-Laccase in culture supernatants after 3-day cultivation. C) Ligation test of SilkC-Laccase with SilkT-ADF3-SpyC. SDS-PAGE image and Western blot of precursors and ligation products with anti-His antibody. M stands for marker. Red arrows depict for the ligation products of added precursors. Theoretical molecular weight of proteins: SilkC-Laccase is 74.1 kDa, SpyC-ADF3-SilkT is 62.3 kDa.

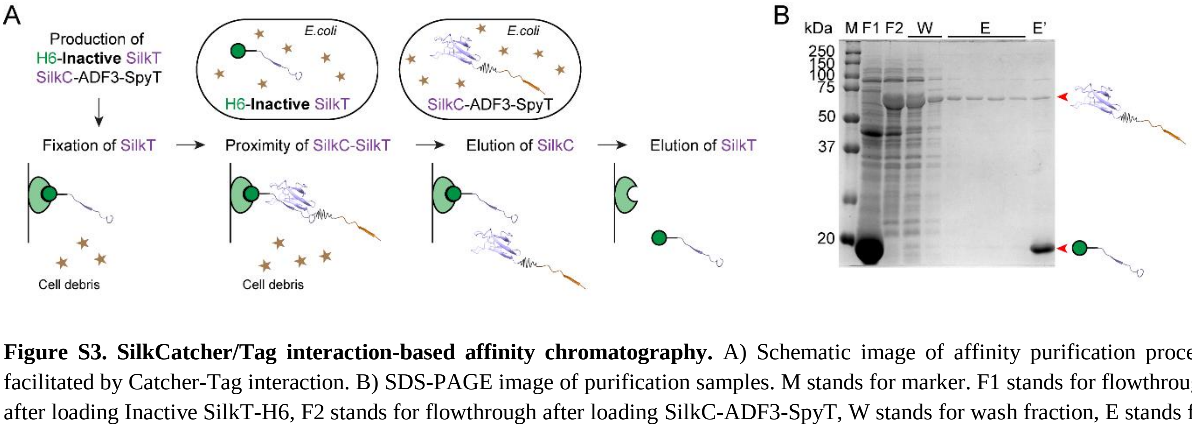

Figure S3. SilkCatcher/Tag interaction-based affinity chromatography. A) Schematic image of affinity purification process facilitated by Catcher-Tag interaction. B) SDS-PAGE image of purification samples. M stands for marker. F1 stands for flowthrough after loading Inactive SilkT-H6, F2 stands for flowthrough after loading SilkC-ADF3-SpyT, W stands for wash fraction, E stands for elution of SilkC-ADF3-SpyT using elution buffer E, E' stands for elution of Inactive SilkT-H6 using elution buffer E'. Theoretical molecular weight of proteins: SilkC-ADF3-SpyT is 57.446 kDa and Inactive SilkT-H6 is 21.081 kDa.

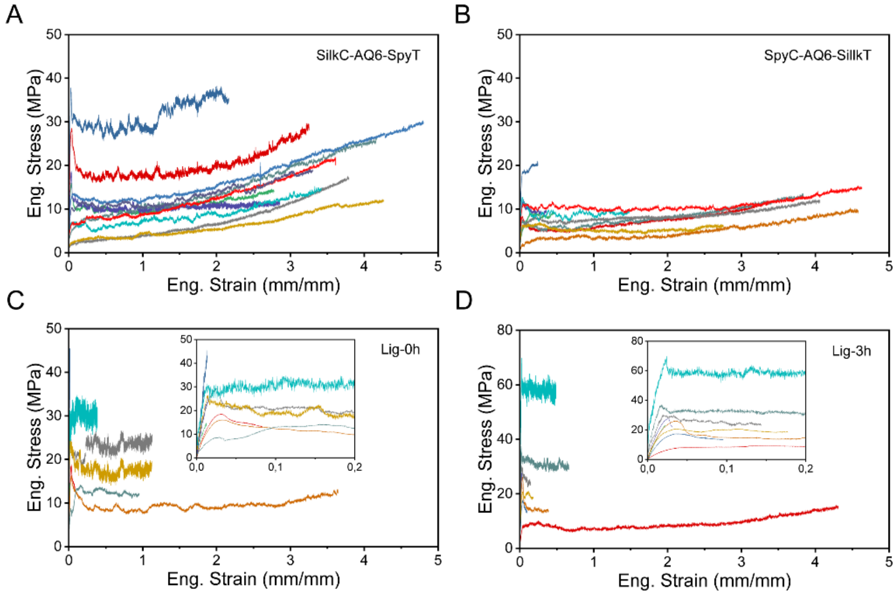

Figure S4. Mechanical properties of fibers. A) Representative tensile test curves of SilkC-ADF3-SpyT fibers triggered in 4 M ammonium acetate solution, pH 7.0. n = 11. B) Representative tensile test curves of SpyC-ADF3-SilkT fibers triggered in 4 M ammonium acetate solution, pH 7.0. n = 10. C) Representative tensile test curves of 0h-polymerized fibers triggered in 4 M ammonium acetate solution, pH 7.0. n = 9. D) Representative tensile test curves of 3h-polymerized fibers triggered in 4 M ammonium acetate solution, pH 7.0. n = 9.

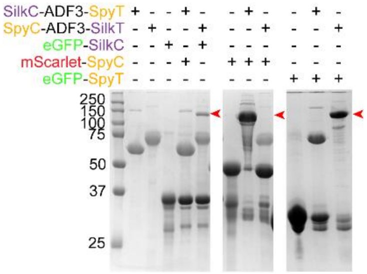

Figure S5. SDS-PAGE images of the ligation reaction between host and client proteins used for recruitment. Theoretical molecular weights of the proteins: SilkC-ADF3-SpyT is 57.4 kDa, SpyC-ADF3-SilkT is 62.3 kDa, eGFP-SilkC is 37.6 kDa, mScarlet-SpyC is 41.2 kDa, and eGFP-SpyT is 30.7kDa. Red arrows depict for the ligation products of added precursors.

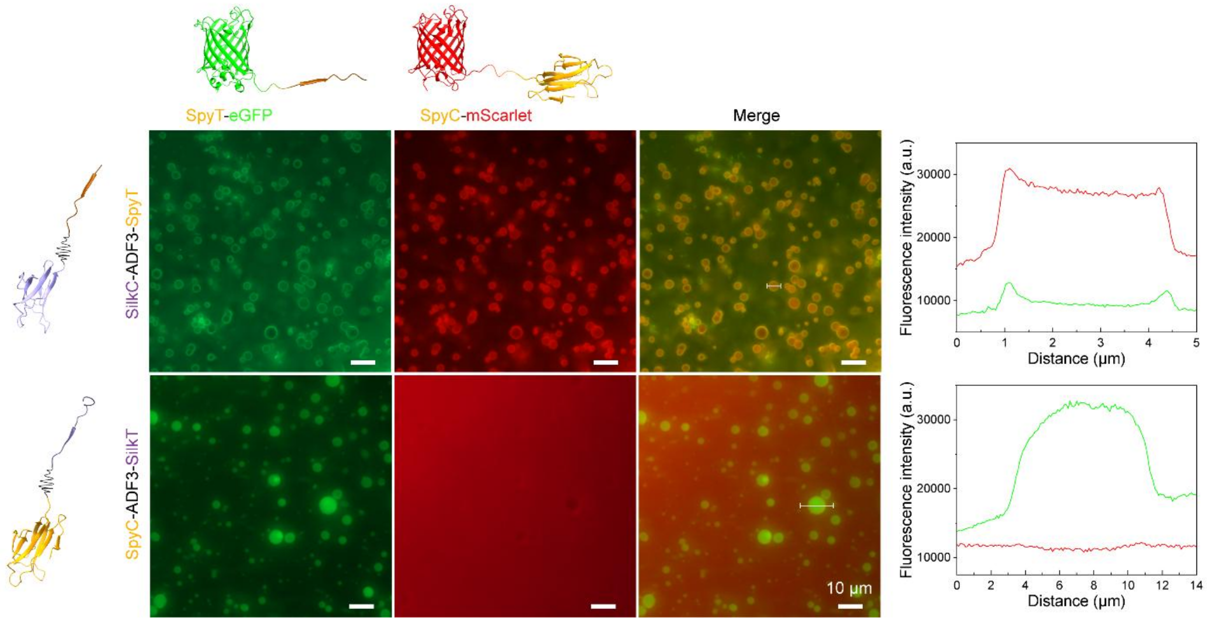

Figure S6. Colocalization fluorescence microscopy images of liquid droplets of different host coacervate, SilkC-ADF3-SpyT and SpyC-ADF3-SilkT, and different client proteins, SpyC-mScarlet and SpyT-eGFP. Scale bar is 10µm.

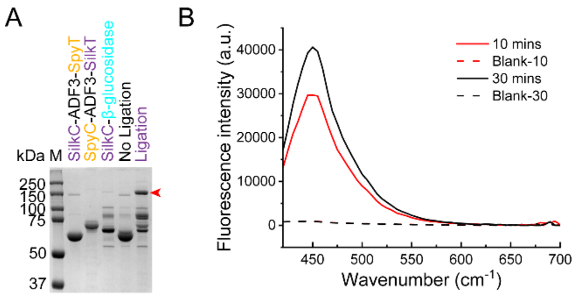

Figure S7. A) SDS-PAGE analysis of ligation between recombinant spider-silk like proteins and SilkC-β-glucosidase. 'No ligation' lane is ligation product of SilkC-ADF3-SpyT and SilkC-β-glucosidase, while 'Ligation' lane is ligation product of SpyC-ADF3-SilkT and SilkC-β-glucosidase. M stands for marker. Theoretical molecular weight of three proteins: SilkC-ADF3-SpyT is 57.4 kDa, SpyC-ADF3-SilkT is 62.3 kDa, and SilkC-β-glucosidase is 68.8 kDa. B) 4-MUG activity assay of SilkC-β-glucosidase fusion protein.

Reference

[1] L. Lemetti, A. Scacchi, Y. Yin, M. Shen, M. B. Linder, M. Sammalkorpi, A. S. Aranko, *Biomacromolecules* 2022, *23*, 3142.
[2] R. Fan, J. Hakanpää, K. Elfving, H. Taberman, M. B. Linder, A. S. Aranko, *Angewandte Chemie - International Edition* 2023, *62*, e202216371.
[3] J. Lin-Cereghino, W. W. Wong, S. Xiong, W. Giang, L. T. Luong, J. Vu, S. D. Johnson, G. P. Lin-Cereghino, *Biotechniques* 2005, *38*, 44.
[4] N. Nishibori, K. Masaki, H. Tsuchioka, T. Fujii, H. Iefuji, *J Biosci Bioeng* 2013, *115*, 394.
[5] K. Hildén, M. R. Mäkelä, T. Lundell, J. Kuuskeri, A. Chernykh, L. Golovleva, D. B. Archer, A. Hatakka, *Applied Microbiology and Biotechnology 2012 97:4* 2012, *97*, 1589.
[6] T. Kenzom, P. Srivastava, S. Mishra, *Appl Environ Microbiol* 2014, *80*, 7484.
[7] G. Greco, B. Schmuck, S. K. Jalali, N. M. Pugno, A. Rising, *Biophys Rev* 2023, *4*, 031301.
